# Reaction-diffusion memory unit: Modeling of sensitization, habituation and dishabituation in the brain

**DOI:** 10.1371/journal.pone.0225169

**Published:** 2019-12-05

**Authors:** Matthew M. Carnaghi, Joseph M. Starobin

**Affiliations:** Department of Nanoscience, Joint School of Nanoscience and Nanoengineering, University of North Carolina at Greensboro, Greensboro, North Carolina, United States of America; University of Antwerp, BELGIUM

## Abstract

We propose a novel approach to investigate the effects of sensitization, habituation and dishabituation in the brain using the analysis of the reaction-diffusion memory unit (RDMU). This unit consists of Morris-Lecar-type sensory, motor, interneuron and two input excitable cables, linked by four synapses with adjustable strength defined by Hebbian rules. Stimulation of the sensory neuron through the first input cable causes sensitization by activating two excitatory synapses, *C*_*1*_ and *C*_*2*_, connected to the interneuron and motor neuron, respectively. In turn, the stimulation of the interneuron causes habituation through the activation of inhibitory synapse *C*_*3*_. Likewise, dishabituation is caused through the activation of another inhibitory synapse *C*_*4*_. We have determined sensitization-habituation (BSH) and habituation-dishabituation (BHDH) boundaries as functions between synaptic strengths *C*_*2*_ and *C*_*3*_ at various strengths of *C*_*1*_ and *C*_*4*_. When BSH and BHDH curves shift towards larger values of *C*_*2*_, the RDMU can be easily inhibited. On the contrary, the RDMU can be easily sensitized or dishabituated if BSH and BHDH curves shift towards smaller values of *C*_*2*_. Our numerical simulations readily demonstrate that higher values of the Morris-Lecar relaxation parameter, greater leakage and potassium conductances, reduced length of the interneuron, and higher values of *C*_*1*_ all result in easier habituation of the RDMU. In contrast, we found that at higher values of *C*_*4*_ the RDMU becomes significantly more prone to dishabituation. Based on these simulations one can quantify BSH and BHDH curve shifts and relate them to particular neural outcomes.

## Introduction

The formation of memory has been linked to long-term potentiation (LTP) and long-term depression (LTD), or the lasting increase or decrease of the strength of synaptic connections [1], [2]. Through LTP and LTD, information can be stored and behavioral patterns can become fixed in the brain. LTP and LTD occur after a period of learning, which is dominated by sensitization and habituation [2].

Sensitization and habituation are two basic processes in memory that are controlled by the strength of synaptic connections. Sensitization increases the probability that a given stimulus will produce a downstream transmembrane potential by increasing the connectivity of excitatory synapses while habituation decreases the probability of an transmembrane potential by increasing the connectivity of inhibitory synapses [3], [4]. This has been demonstrated in experiments on the gill withdrawal reflex in *Aplysia californica*, where repeated stimulation caused a prolonged withdrawal of the gill [5], [6].

The process of altering behavior based on changes in synaptic connection strengths is known as synaptic plasticity and is considered to be the underlying mechanism of the formation of memory [5], [7]. So far, the main stream approach to model the formation of memory was based on the threshold models of individual neurons [8], [9], [10]. Nevertheless, these models did not reflect physiological reaction-diffusion mechanisms which are responsible for the conduction of excitation in the neuronal environment. A recent effort to incorporate reaction-diffusion effects to quantify changes in the synaptic strength of isolated biological synapses [11] and synaptic-like memristive elements [12] was a step in the right direction. However, it still did not help to elucidate the reaction-diffusion origin of sensitization and habituation.

Another way to account for the spatial distribution of neuronal structures is the introduction of the concept of a meta-neuron. A meta-neuron consists of a relatively small group of several tens of neurons, which may be collectively involved in a particular macroscopic function. Such a structure includes Hodgkin-Huxley axons with added synaptic connections described by a set of gating equations [13]. In general, this approach may be considered as an adequate tool to describe large neuronal clusters, yet it entirely ignores the essential structural details which govern the balance between sensitization and habituation needed to process the information by a particular memory unit.

A typical example of sensitization and habituation can be seen in the startle response in zebrafish. The startle circuit is comprised of an auditory sensory neuron, a Mauthner cell (motor neuron) that triggers startle movement, and an inhibitory neuron that prevents familiar stimuli from triggering a startle response [14], [15]. The auditory neuron connects to both the inhibitory neuron and Mauthner cell with two excitatory synapse regions. The inhibitory neuron also connects to the Mauthner cell with an inhibitory synapse region. As a new incoming stimulus is repeated, the excitatory synapses connecting to the inhibitory neuron strengthen, and inhibitory synapses connecting to the Mauthner cell strengthen as well, resulting in less frequent triggering of the startle response. Although the described above circuit may be useful for explanation of a simple startle response in zebrafish [14], it lacks sufficient complexity to relate to the abrupt disappearance of habituation (dishabituation) which, according to Groves and Thompson dual-process theory, occurs even if the strength of excitatory synapses in the sensory neuron remains constant [4].

More complex examples of habituation are described in Sokolov’s comparator and Ramaswami’s negative-image models [16], [17]. The system for the formation of the model in Sokolov’s approach inhibits the excitatory process for recognized stimuli. When presented with unfamiliar stimuli, this inhibition ceases, resulting in dishabituation. Similarly, dishabituation may also occur in the negative-image model by inhibiting the negative image that negates incoming stimuli.

**Fig 1:**
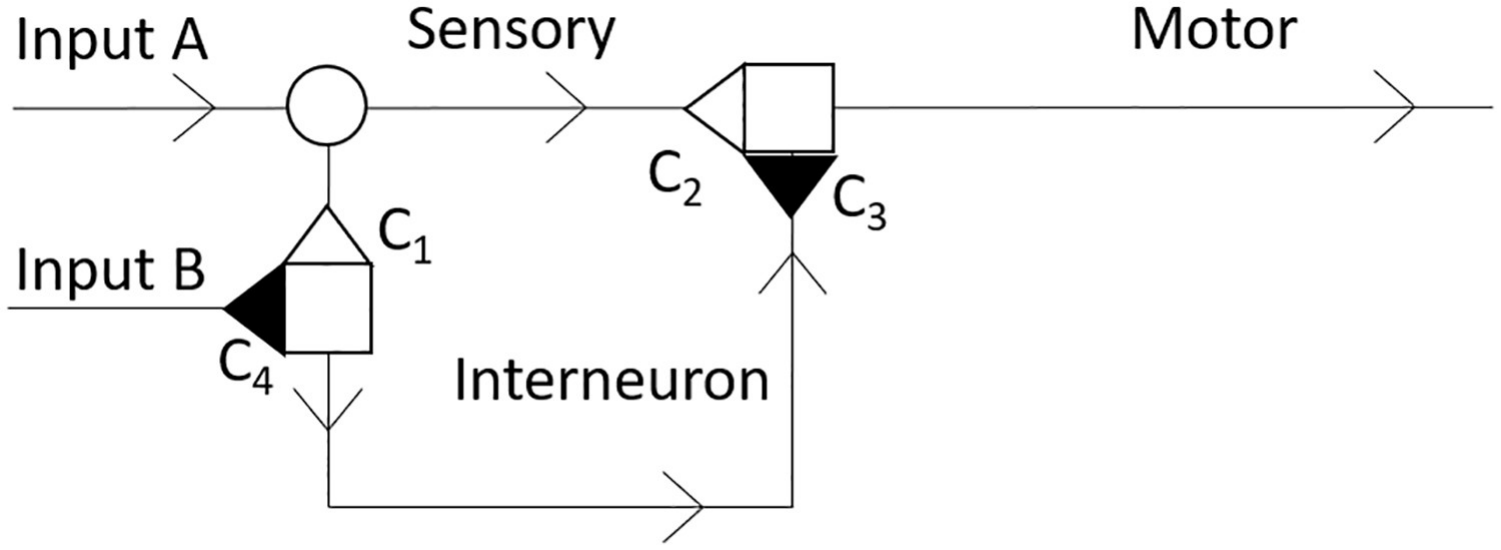
Schematic diagram which illustrates the composition of the RDMU with two stimulating inputs A and B. Excitatory synapses *C*_*1*_ and *C*_*2*_ are shown as empty triangles. Inhibitory synapses *C*_*3*_ and *C*_*4*_ are represented by filled-in triangles. Synaptic junctions are denoted by empty squares and the neuronal branching point is marked with an empty circle. Arrows represent the direction of propagation of transmembrane potentials. The portion of the RDMU between *C*_*1*_ and *C*_*3*_ is the interneuron.

Based on these examples we propose a novel method based on the reaction-diffusion approach that is capable of quantifying combined effects of sensitization, habituation and dishabituation by connecting just a few axons with several synapses of adjustable strength. This model incorporates a circuit of three Morris-Lecar-type neurons [18] linked by four synapses defined by Hebbian synaptic strength rules [19]. The circuit is connected to two distinct Morris-Lecar-type input cables to allow a separate stimulation of sensory and inhibitory neurons. We will further refer to this circuit as the reaction-diffusion memory unit (RDMU, Fig 1).

Unlike conventional spiking threshold models, our reaction-diffusion approach eliminates the need for the use of purely phenomenological temporal delays associated with propagation of excitation from one neuron to another [20], [21]. Instead, these delays form naturally as a result of spatio-temporal evolution of excitation waves under the influence of different rates of cellular membrane polarization and re-polarization processes, various neuronal lengths, and altered strengths of synaptic connections between different neuronal fibers.

## Model

**Fig 2:**
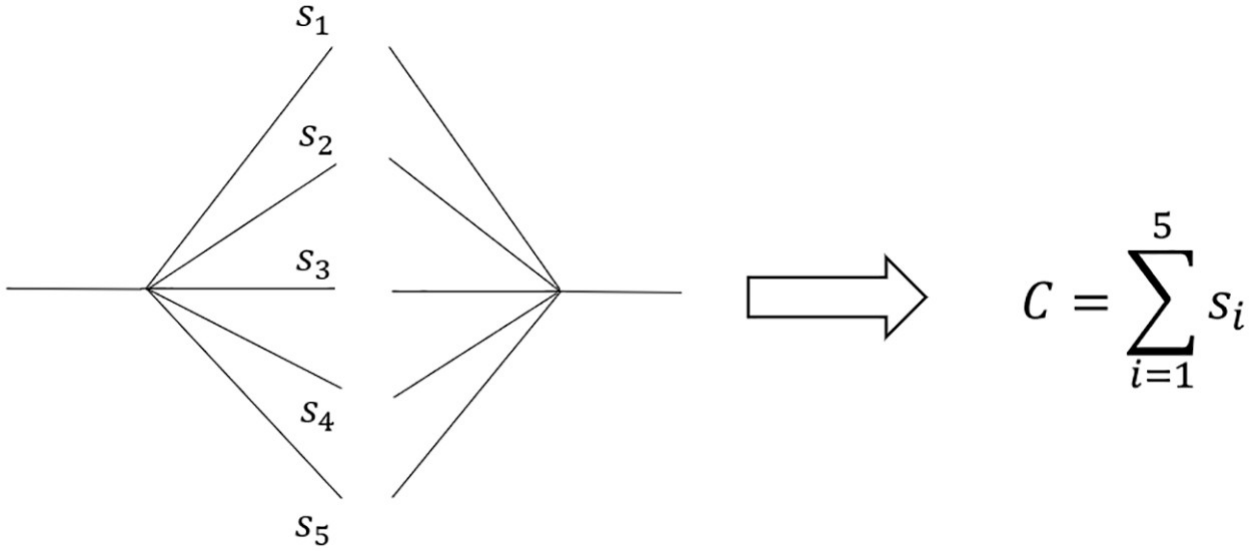
The summation of weights of individual synaptic connections to a conglomerate synapse *C*.

It is possible to illustrate each individual synaptic connection as shown in Fig 2. To quantify cases with an arbitrary number of such connections, we define a variable *C* as a conglomerate synapse given by the sum in Eq (1)

$$C=\sum_{i=1}^{n}s_{i}$$ (1)

where excitatory and inhibitory synaptic wieghts *s*_*i*_ between two specific neurons have positive and negative signs, respectively. The sign of the conglomerate synapse *C* tells whether the net effect of all connections in the bundle is excitatory (+) or inhibitory (-) (Fig 2).

Using synaptic connections determined by Eq (1), we can link neurons to elucidate the processes of sensitization, habituation, and dishabituation (Fig 1). In this figure, the initial propagation of the transmembrane potential is provided by stimulating inputs A and B. The further evolution of the wave from input A causes sensitization through the passage of excitatory synapse *C*_*2*_. The wave also propagates to the interneuron through excitatory synapse *C*_*1*_, and produces habituation via inhibitory synapse *C*_*3*_. The evolution of the wave from input B causes dishabituation by passing through inhibitory synapse *C*_*4*_.

We study the RDMU mathematically by using a Morris-Lecar model with incorporated Hebbian conditions at the synaptic junctions, no flux boundary conditions at the ends of neurons, and additional diffusion terms at the sensory neuron’s branching point [18] (Fig 1). The equations for the Morris-Lecar model are as follows:

$$C_{0}\frac{\partial v}{\partial t}=-g_{L}(v-v_{L})-M_{\infty }g_{Ca}(v-v_{Ca})-g_{K}w(v-v_{K})+D\frac{\partial^{2}v}{\partial x^{2}}+F(t)$$ (2)

$$\frac{\partial w}{\partial t}=\frac{{(W}_{\infty }-w)}{\tau }$$ (3)

$$M_{\infty }=\frac{1}{2}(1+\text{tanh}\left(\frac{v-v_{1}}{v_{2}}\right))$$ (4)

$$W_{\infty }=\frac{1}{2}(1+\text{tanh}\left(\frac{v-v_{3}}{v_{4}}\right))$$ (5)

$$\tau =\frac{1}{\phi }\text{sech}\left(\frac{v-v_{3}}{{2v}_{4}}\right)$$ (6)

$$F(t)=\{\begin{array}{c} I,\;t\leq t_{dur}\\ 0,\;t>t_{dur} \end{array}$$ (7)

where *I* and *t*_*dur*_ are the amplitude of the external current (stimulus) and its duration, respectively. Variables *v* and *w* represent the transmembrane voltage and dimensionless gating variable corresponding to the inhibitory response of the potassium channels. Parameters *v*_*L*_, *v*_*Ca*_, and *v*_*K*_ are equilibrium potentials for leakage, calcium, and potassium currents, respectively. Factors *M*_*∞*_ and *W*_*∞*_ are dimensionless constants which are determined by regulating voltages *v*_*1*_, *v*_*2*_, *v*_*3*_, and *v*_*4*_ [22].

By introducing specific time and spatial scales, one can define a set of dimensionless variables as follows:

$$v^{*}=\frac{v}{v_{Ca}},\;v_{i}^{*}=\frac{v_{i}}{v_{Ca}},\;i=L,K,Ca,1,2,3,4$$ (8)

$$t^{*}=t\frac{g_{Ca}}{C_{0}}$$ (9)

$$x^{*}=\frac{x}{L_{0}}$$ (10)

$$L_{D}=\sqrt{\frac{D}{g_{Ca}}}$$ (11)

$$g_{i}^{*}=\frac{g_{i}}{g_{Ca}},\;i=L,K,Ca$$ (12)

$$\phi^{*}=\phi \frac{C_{0}}{g_{Ca}}$$ (13)

Here v*, t*, and x* are dimensionless variables for transmembrane potential, time and spatial variables. Parameters *g*_L_* and *g*_K_* refer to dimensionless leakage and potassium conductance, respectively. Parameter g_Ca_*, which determines dimensionless calcium conductance, is equal to one. The value of *L*_*D*_ corresponds to the diffusion length. The value of *L*_*0*_ is the length of the main section of the RDMU, which is equal to the sum of the lengths of the sensory and motor neurons. The scales are given as follows: *C*_*0*_ = 10μF, *v*_*Ca*_ = 100mV, *D* = 1μS•cm^2^, *g*_*Ca*_ = 10mS, and *L*_*0*_ = 1mm.

For simplicity, we will further refer to dimensionless variables as *v*, *w*, *t*, *x*, *g*_*K*_, *v*_*K*_, etc., continuing with dimensionless Morris-Lecar equations in the following way:

$$\frac{\partial v}{\partial t}=F(t)-g_{L}(v-v_{L})-M_{\infty }(v-v_{Ca})-g_{K}w(v-v_{K})+{\left(\frac{L_{D}}{L_{0}}\right)}^{2}\frac{\partial^{2}v}{\partial x^{2}}$$ (14)

$$\frac{\partial w}{\partial t}=\frac{\left(W_{\infty }-w\right)}{\tau }$$ (15)

$$M_{\infty }=\frac{1}{2}(1+\text{tanh}\left(\frac{v-v_{1}}{v_{2}}\right))$$ (16)

$$W_{\infty }=\frac{1}{2}(1+\text{tanh}\left(\frac{v-v_{3}}{v_{4}}\right))$$ (17)

$$\tau =\frac{1}{\phi }\text{sech}\left(\frac{v-v_{3}}{{2v}_{4}}\right)$$ (18)

Table 1 summarizes the model dimensionless parameters which, unless stated otherwise, were used in all numerical experiments. These values are based on the dimensional values from [22].

**Table 1:** The dimensionless parameters used to solve model Eqs (14)–(22).

*ϕ*	*g*_*Ca*_	*g*_*K*_	*g*_*L*_	*v*_*Ca*_	*v*_*K*_	*v*_*L*_	*v*_1_	*v*_2_	*v*_3_	*v*_4_	*I*	*v*_*o*_	*w*_*o*_	*v*^*Thr*^
0.017	1	1.8	0.45	1	-0.84	-0.6	-0.012	0.18	0.02	0.30	1	-0.58	0.0177	-0.225

A set of boundary conditions for Eqs (14)–(18) includes no-flux conditions at the ends of the neurons (Eq (19)), and Hebbian links between the pre- and postsynaptic values of transmembrane potential at each of the RDMU synapses (Eq (20)) [19].

$$\frac{\partial v}{\partial x}=0$$ (19)

$$v_{A,post}-v_{o}=C_{2}(v_{A,pre}-v_{o})+C_{3}(v_{B,pre}-v_{o})$$ (20A)

$$v_{B,post}-v_{o}=C_{1}(v_{A,pre}-v_{o})+C_{4}(v_{B,pre}-v_{o})$$ (20B)

**Fig 3:**
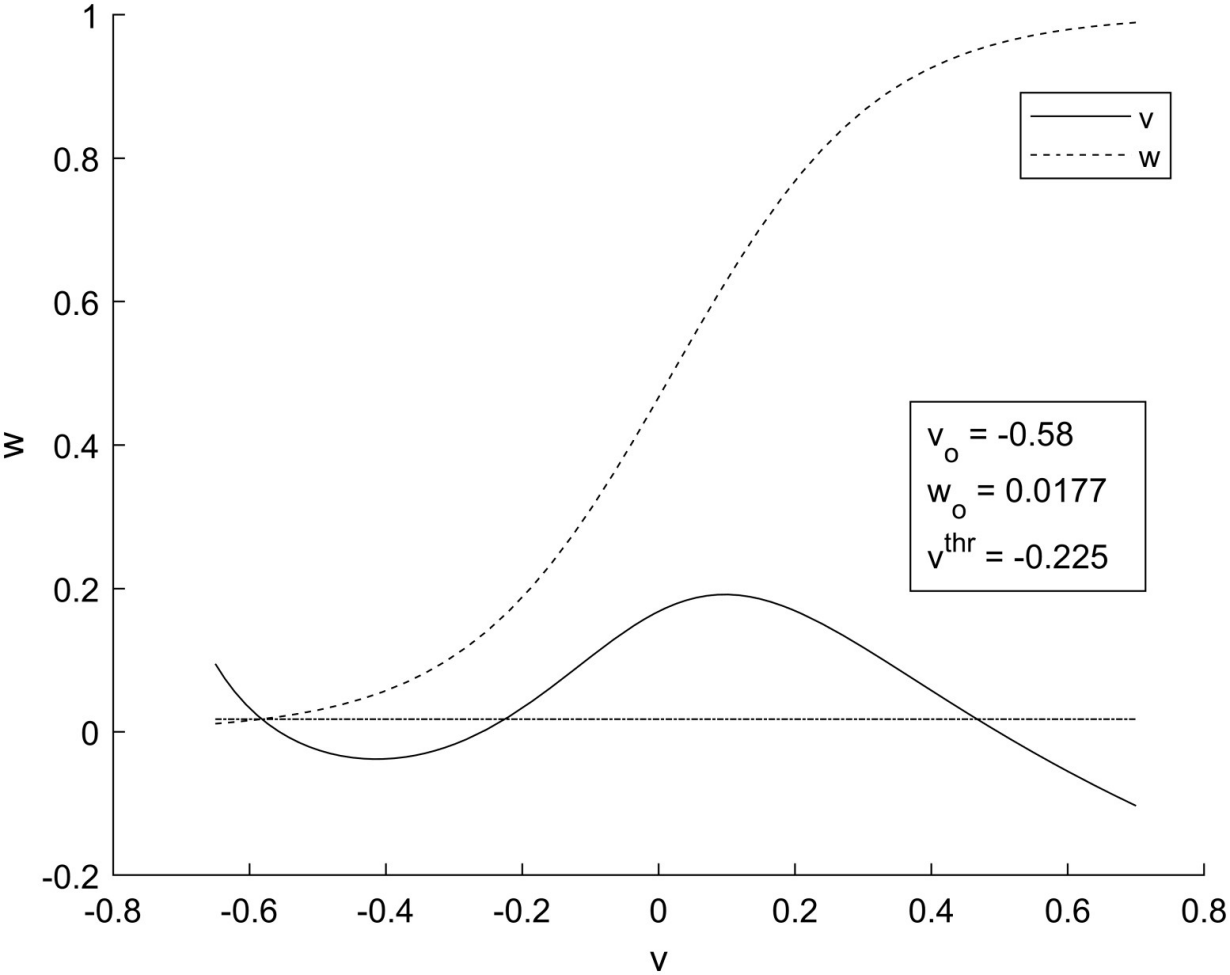
Phase portrait for the Morris-Lecar model. The solid line is the null-cline for *v* and the dashed line is the null-cline for *w*. The equilibrium values for *v* and *w* occur at the intersection of null-clines. The horizontal dot-dashed line has a value of *w*_*o*_ for all *v*, and is used to find *v*^*thr*^, which is the second intersection of *v* and *w*_*o*_.

Here *C*_*i*_, *v*_*i*,*pre*_, *v*_*i*,*post*_, and *v*_*o*_ are synaptic strengths, pre- and post-synaptic potentials of the *i*^*th*^ synapse and resting value of transmembrane potentials, respectively. The first of Eq (20) describes the cumulative post-synaptic action of adjacent excitatory and inhibitory synapses *C*_*2*_ and *C*_*3*_, located at the beginning of the motor neuron. The second part describes the cumulative post-synaptic action of adjacent excitatory and inhibitory synapses *C*_*1*_ and *C*_*4*_, located at the beginning of the interneuron. The resting transmembrane potential, *v*_*o*_, as well as the resting value of the recovery variable, *w*_*o*_, are determined by the intersection of null-clines of the system of Eqs (14) and (15). The null-clines also define the excitation threshold, *v*^*thr*^, as shown in Fig 3.

To complete the formulation of synaptic conditions one needs an additional boundary condition to warrant that each synapse acts as a unidirectional gate which prevents the backward flow of transmembrane potentials. This condition is defined by Eq (22):

$${\frac{\partial v}{\partial x}|}_{x=x^{-}}=0$$ (21)

Here *x*^−^ is upstream with respect to the direction of the synaptic current.

Finally, we analyze the branching point located at the end of input A, where the sensory axon diverges (Fig 1). At this point we need to modify Eq (14) and consider two diffusion terms to account for cumulative two-dimensional effects comprised of two one-dimensional diffusion processes in the first (*x*) and the second (*y*) neuron branches, respectively:

$$\frac{\partial v}{\partial t}=F(t)-g_{L}(v-v_{L})-M_{\infty }(v-v_{Ca})-g_{K}w(v-v_{K})+{\left(\frac{L_{D}}{L_{0}}\right)}^{2}(\frac{\partial^{2}v}{\partial x^{2}}+\frac{\partial^{2}v}{\partial y^{2}})$$ (22)

The rationale for considering a linear steady-state Hebbian rule (Eq (20)) is based upon the observation that behavioral and, to some extent, cognitive memories are associated with neural oscillations within theta and partial gamma ranges below 20 Hz [23], [24]. Under these conditions one can consider only isolated stimulating currents (Eq (7)) applied to the sensory neuron shown in Fig 1. Indeed, the transmembrane potentials induced by neuronal spikes in the hippocampus are on average 1-3ms in duration [25] and the intervals between successive spikes at frequencies below 20Hz are greater than 50ms. Therefore, the temporal evolution of the transmembrane potential resulted from a previous neuronal spike becomes completed well before the initiation of the next spiking activity. Accordingly, propagation of the transmembrane potentials in the RDMU branches evolves into transmission of the steady-state solitary pulses. Finally, since the propagation of transmembrane potentials is steady-state, a temporal derivative term in the Hebbian rule [19] can be omitted and resulting steady-state Hebbian links can be expressed as linear algebraic relations described by Eq (20).

### Numerical method and system parameters

The system of Eqs (14)–(22) was solved numerically using an explicit finite difference method (See S1 Appendix). The dimensionless time and spatial steps were *Δt* = 2.5×10^−5^ and *Δx* = .01 for all experiments, respectively. Parameter ${\left(\frac{L_{D}}{L_{0}}\right)}^{2}$ in Eqs (14) and (22) was set to 0.01.

**Fig 4:**
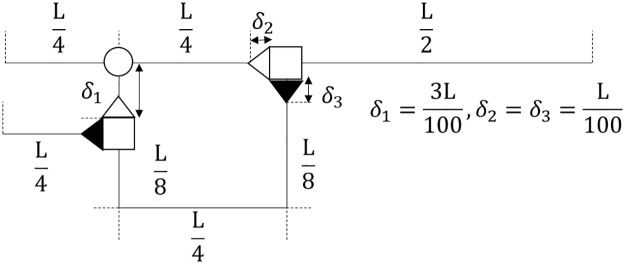
The diagram which depicts the spatial scales of the RDMU.

Unless stated otherwise, the sensory neuron and both inputs spanned 25 spatial intervals each, while the motor neuron and interneuron individually consisted of 50 spatial intervals (Fig 4). At the initial time *t* = 0 an external stimulus *I* of amplitude one was applied for a duration of 5×10^4^*Δt* to nodes one through fifteen located at the beginning of inputs A and B.

**Fig 5:**
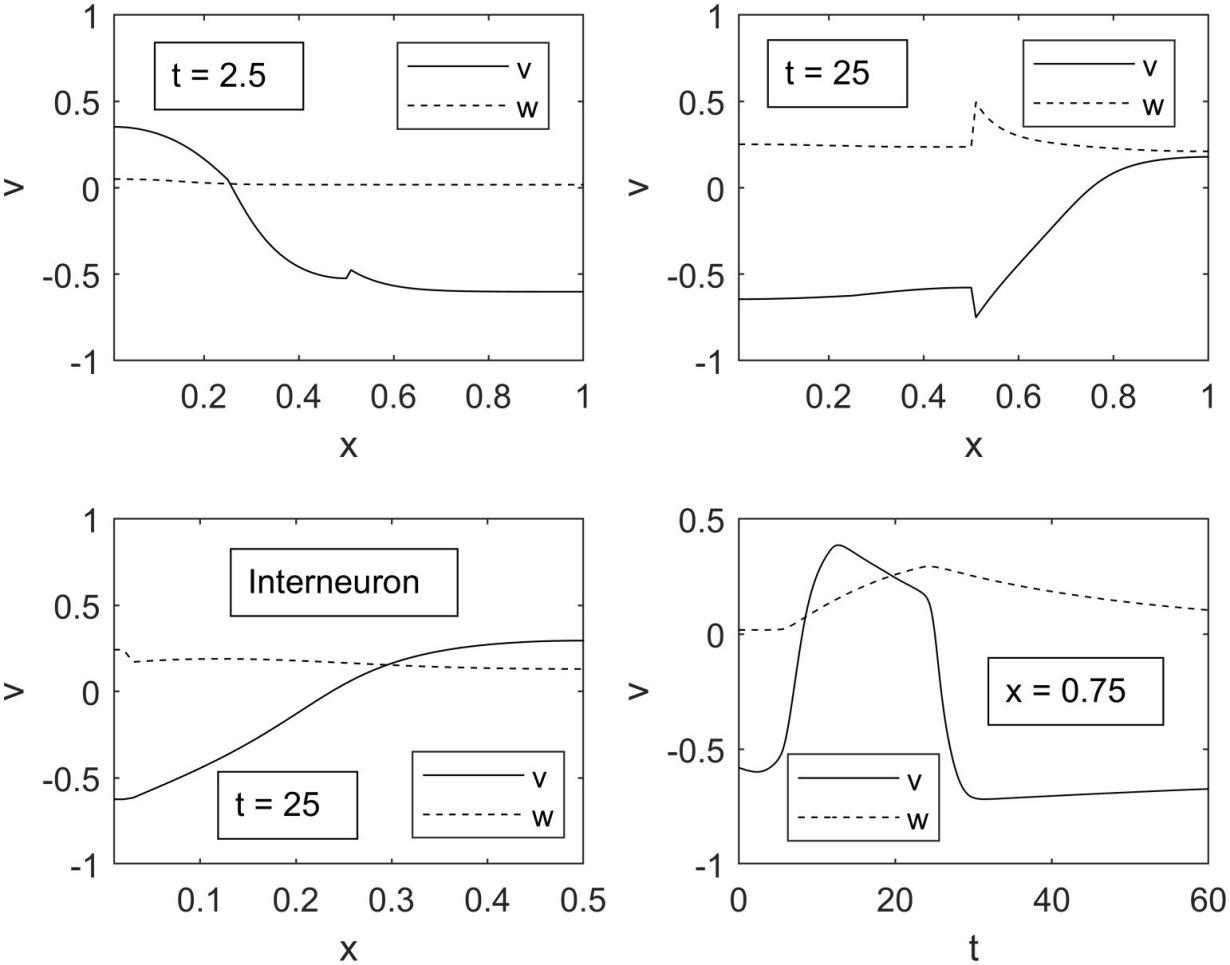
Transmembrane potential *v* and gating variable *w* as functions of spatial variable *x*. Upper panels show a spatial evolution of the excitation pulse in the sensory and motor neurons in the interval of time between 2.5 and 25. Lower left panel shows progression of the excitation pulse in the interneuron at time 25. Lower right panel illustrates temporal evolution of excitation at *x* = 0.75. Parameters *C*_*1*_, *C*_*2*_, and *C*_*3*_ are equal to 1, 1.8, and -0.2, respectively, for ideal propagation through all regions.

Taking into consideration that the speed of transmembrane potentials in the brain is on average greater than 10m/s, parameters in Table 1 were set to reflect that the width of the excitation wave is much longer than a one millimeter total length of the sensory and motor neurons [26], [27]. Typical spatial and temporal evolutions of such waves are depicted in Fig 5.

### Experimental protocol for numerical simulations

We studied the propagation of solitary pulses originated by identical input stimuli, *I*, applied to both inputs of the RDMU. A series of numerical simulations has been performed in order to evaluate the RDMU’s ability to reproduce the processes of sensitization, habituation and dishabituation. Depending on the values of synaptic strengths *C*_*1*_, *C*_*2*_, *C*_*3*_, and *C*_*4*_, the input stimuli propagated to the motor neuron and originated either sub-threshold or over-threshold responses, thus signifying the initiation of processes of sensitization, habituation and dishabituation.

The synaptic strength boundary between sensitization and habituation (BSH) was computed iteratively with the value of *C*_*4*_ fixed at zero. At fixed values of *C*_*2*_ we incrementally adjusted the value of *C*_*3*_ until regimes changed from sensitization to habituation, preventing the propagation of the over-threshold stimulus in the motor neuron. After that, values of *C*_*2*_ were increased by a set of sufficiently small increments and the process was repeated until values of *C*_*2*_ were equal to 1.5 or values of *|C*_*3*_*|* exceeded 5, beyond which the BSH and BHDH curves become linearly proportional. The boundary between habituation and dishabituation (BHDH) was calculated in the same manner at different values of *C*_*4*_.

## Results

We determined whether the system was in sensitization, habituation, or dishabituation by comparing the maximum transmembrane potential to a threshold potential shown in Fig 3. The threshold potential was increased by 20% to account for wave propagation decay due to diffusion. BSH and BHDH curves were determined depending on whether the maximum transmembrane potential exceeded the modified threshold or remained below it. It was found that the differences between BSH and BHDH curves measured at 10 *Δx* from the end of the motor neuron and further away (30 *Δx*) did not exceed 5% and 16% at low and high values of *C*_*3*_, respectively. We chose to measure the magnitude of the transmembrane potentials closer to the end of the motor neuron at 10 *Δx*.

### The influence of relaxation parameter, potassium and leakage conductance on shape of the excitation pulse

**Fig 6:**
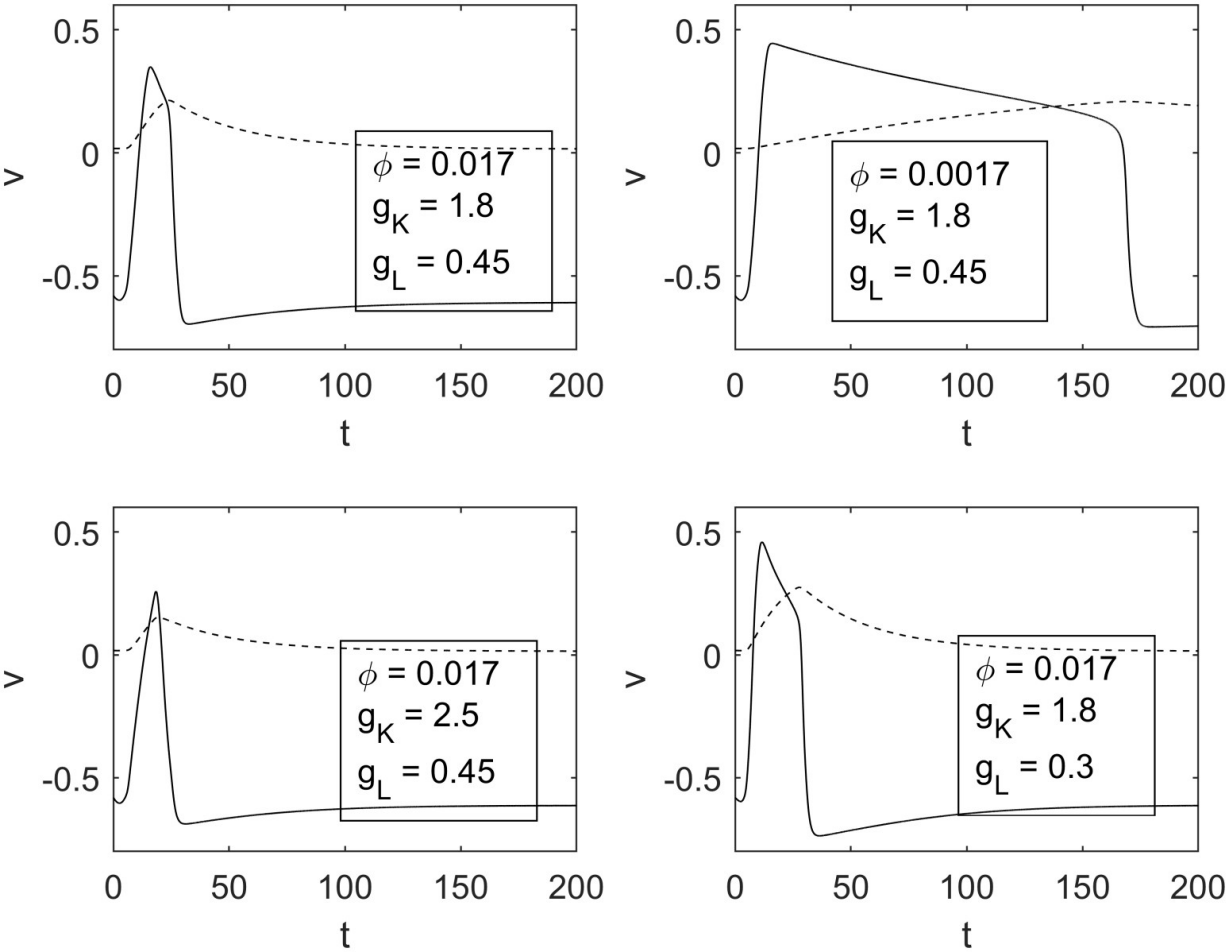
Various transmembrane potentials for different sets of parameters. The top left panel depicts the transmembrane potential for the parameters in Table 1, while the top right, bottom left, and bottom right show transmembrane potentials for decreased *ϕ*, increased *g*_*K*_, and decreased *g*_*L*_, respectively.

One of the main parts of our numerical simulations was focused on investigating the influence of the relaxation parameter *ϕ* and the potassium and leakage conductances on the dynamics of excitation pulses in the RDMU. As expected, we found that the magnitude of *ϕ* significantly affected the rate of relaxation of recovery variable *w*, and therefore invoked considerable changes in the width and speed of the excitation pulse. Potassium and leakage conductances also contributed to changes of the width of the pulse in a noticeable way. Fig 6 demonstrates various shapes of excitation pulses for different parameters *ϕ*, *g*_*K*_ and *g*_*L*_. One can observe that smaller values of *ϕ* cause prolongation of pulses. Similar changes occur due to the decrease of either *g*_*K*_ or *g*_*L*_.

### Boundary between sensitization and habituation. The influence of ϕ, g_K_, g_L_ and C_1_

**Fig 7:**
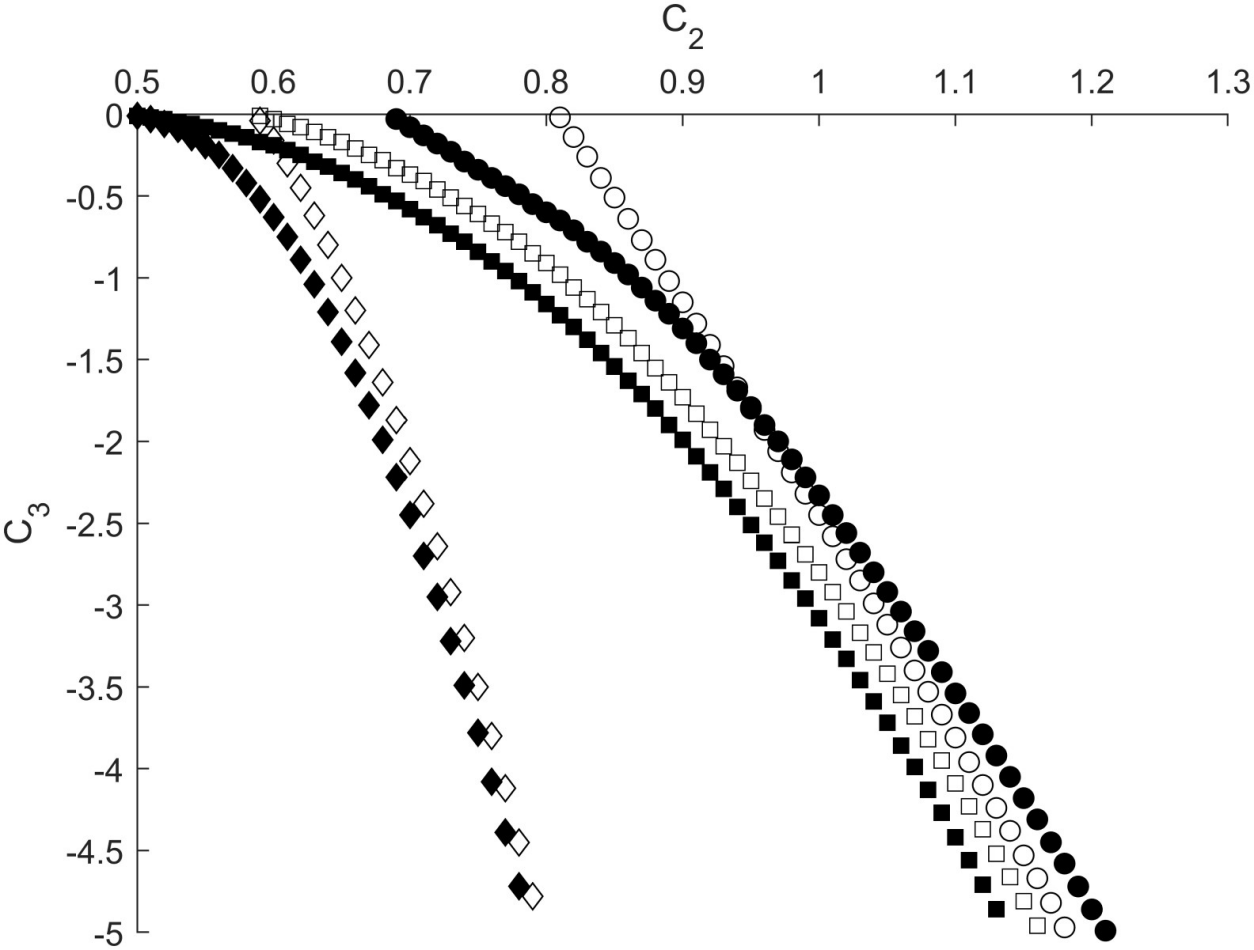
Sensitization-habituation boundaries depicted as dependences of *C*_*3*_ on *C*_*2*_ for different values of *ϕ* and *g*_*L*_ (*g*_*K*_ = 1.8). Open and filled shapes correspond to *ϕ* = 0.017 and *ϕ* = 0.0017, respectively. Parameter *C*_*1*_ is equal to 0.8 for all curves except open and filled diamonds, where *C*_*1*_ = 0.6. Circles correspond to *g*_*L*_ = 0.45 while diamonds and squares relate to *g*_*L*_ = 0.3. Other parameters are fixed at values shown in Table 1.

We performed a series of numerical simulations to study the edge between sensitization and habituation processes in the RDMU, where the propagation of excitation waves is depicted in Fig 6. As shown in Fig 7 the BSH can be adequately described by Eq (23) (see also Table 2)

$$C_{3}=aC_{2}^{b}+c,\;b>1$$ (23)

**Table 2:** Constants a, b, and c for curves depicted in Figs 7 and 8. The values of these constants are determined from Eq (23) using linear regression.

*ϕ*, *g*_*K*_, *g*_*L*_, *C*_1_	*a*	*b*	*c*	Figure/curve shape
0.0017, 2.5, 0.45, 0.8	-3.81	2.76	1.59	8, filled circle
0.0017, 1.8, 0.45, 0.8	-3.37	3.14	1.06	8, filled square
0.0017, 1.8, 0.3, 0.8	-3.39	3.60	0.33	7, filled square
0.017, 1.8, 0.3, 0.8	-3.36	3.47	0.59	7, open square
0.0017, 1.8, 0.3, 0.6	-21.11	5.50	0.57	7, filled diamond
0.017, 1.8, 0.3, 0.6	-18.08	4.08	2.10	7, open diamond

It should be noted that shorter (slower) pulses with higher values of *ϕ* correspond to lower absolute values of the inhibitory synaptic strength *|C*_*3*_*|*, thus indicating that it is easier to counter play an excitatory action of the synapse *C*_*2*_ for higher magnitudes of relaxation parameter *ϕ*. Alternatively, it was found that a decrease of leakage conductance *g*_*L*_ resulted in an opposite shift of BSH towards higher values of *|C*_*3*_*|* associated with greater thresholds required to inhibit the RDMU at any given strength of *C*_*2*_ (Fig 7).

As shown in Fig 1, the excitatory synapse *C*_*1*_ plays a role as some type of a gate which regulates the flow of transmembrane potentials between the sensory and interneuron branches of the RDMU. Specifically, it varies the transmembrane potential’s diffusion flux, and therefore controls the amplitude of the excitation pulse which propagates through the inter-neuronal branch of the RDMU.

**Fig 8:**
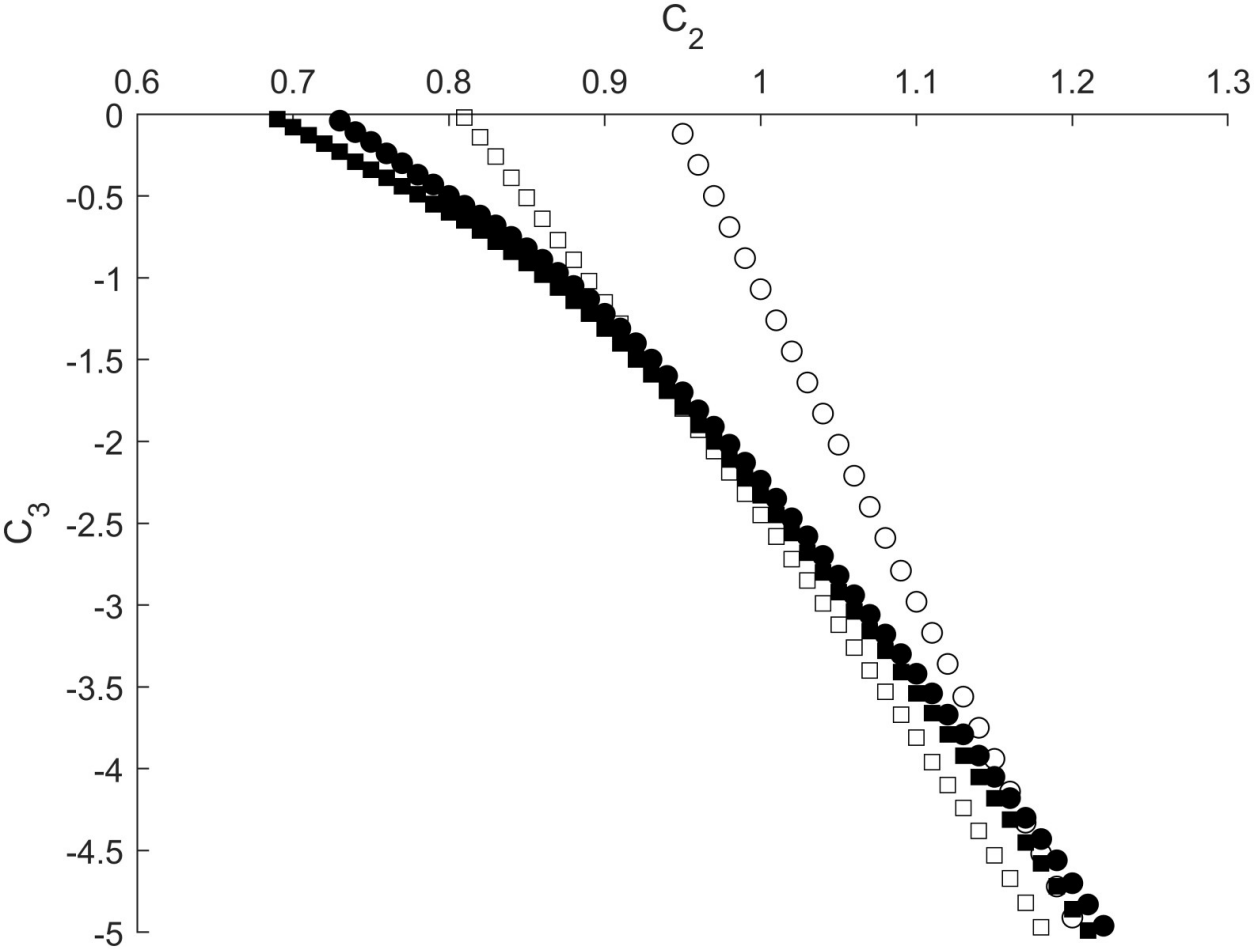
Sensitization-habituation boundaries depicted as dependences of *C*_*3*_ on *C*_*2*_ for different values of *ϕ* and *g*_*K*_ (*g*_*L*_ = 0.45). Open and filled shapes correspond to *ϕ* = 0.017 and *ϕ* = 0.0017, respectively. Circles and squares correspond to *g*_*K*_ = 2.5 and *g*_*k*_ = 1.8, respectively. Other parameters are fixed at values shown in Table 1.

At lower values of *C*_*1*_, as well as in case of lesser *g*_*L*_, we again observed a significant shift of the BSH towards higher values of *|C*_*3*_*|* (Fig 7). Specifically, at *C*_*1*_ = 0.6 and *C*_*2*_ = 0.65 the value of *|C*_*3*_*|* required for the suppression of a pulse in the motor neuron was more than three times greater than a corresponding value of *|C*_*3*_*|* necessary for the suppression of a similar pulse at *C*_*1*_ = 0.8. It should be noted that all BSH curves depicted in Fig 7 are in agreement with approximation (23), since values of *b* are greater than one (Table 2). However, when the relaxation parameter *ϕ* and potassium conductance *g*_*K*_ increase simultaneously the BSH curves turn to nearly directly proportional changes between inhibitory and excitatory synaptic strengths *C*_*2*_ and *C*_*3*_ (Fig 8).

### Boundary between sensitization and habituation. The influence of the length of RDMU branches

It has been demonstrated above that the propagation of excitation waves from the sensory to motor neuron may significantly depend on both the strengths of the excitatory synapses *C*_*1*_ and *C*_*2*_, as well as on the influence of the inhibitory interneuron synaptic connection *C*_*3*_.

**Fig 9:**
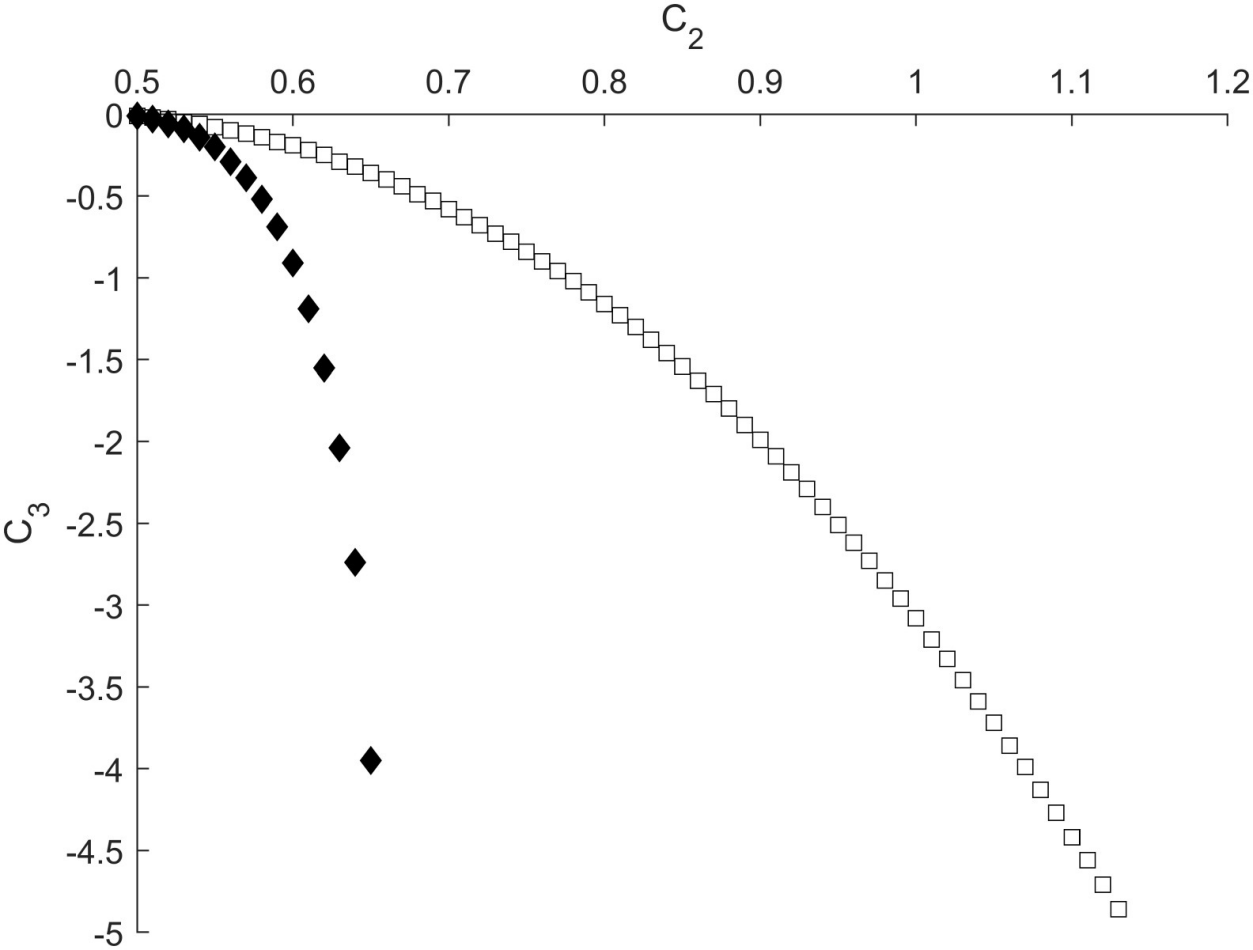
Sensitization-habituation boundaries depicted as dependences of *C*_*3*_ on *C*_*2*_ for different interneuron lengths. Black diamonds denote interneurons with length 0.625 while squares relate to interneurons with length 0.5. Parameters *ϕ* and *g*_*L*_ are equal to 0.0017 and 0.3, respectively. Other parameters are fixed at values shown in Table 1.

We also found that the lengths of the RDMU neurons can be additional important contributors into the balance between habituation and sensitization. Accordingly, the larger ratio of the interneuron’s length to the total length of the sensory and motor neurons results in more significant shift of the BSH curve to the left, making it more difficult to inhibit the RDMU even at smaller values of *C*_*2*_ (Fig 9).

### Boundary between habituation and dishabituation. The influence of C_4_

To calculate the BHDH curves, we applied two stimuli through inputs A and B. As shown in Fig 1, input A connects directly to the sensory neuron while input B connects to the interneuron through inhibitory synapse *C*_*4*_. In this manner, *C*_*4*_ affects the BHDH curves by decreasing the responsiveness of the interneuron.

**Fig 10:**
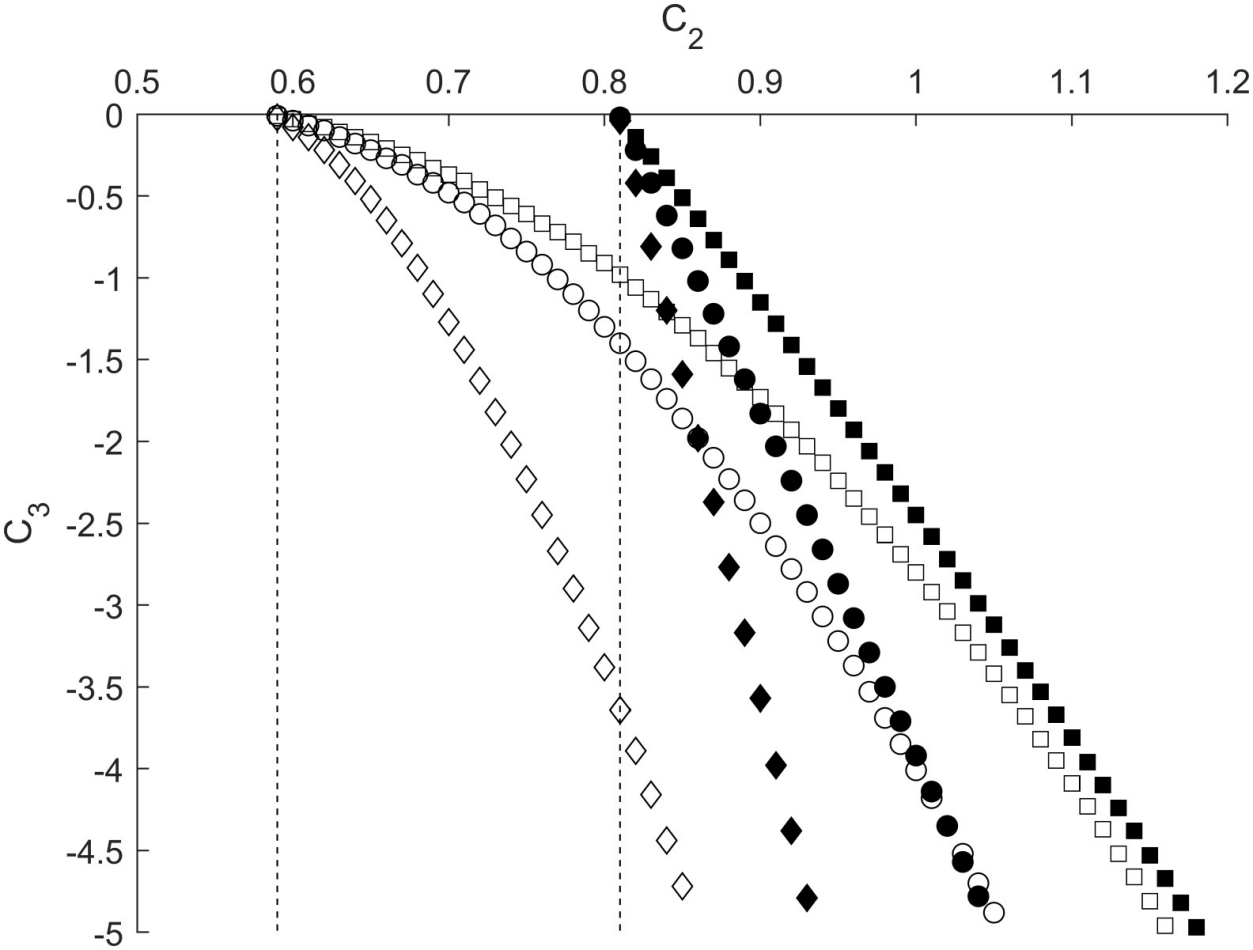
Habituation-dishabituation boundaries depicted as dependences of *C*_*3*_ on *C*_*2*_ for different values of *C*_*4*_. Filled shapes represent *g*_*L*_ equal to 0.45 while empty shapes represent *g*_*L*_ equal to 0.3. Squares, circles, and diamonds stand for *C*_*4*_ equal to 0, 0.05, and 0.15, respectively. Dashed lines correspond to *C*_*4*_ equal to 0.225. Other parameters are fixed at values shown in Table 1.

We found that increasing the strength of *C*_*4*_ resulted in a shift of BHDH curves towards smaller values of *C*_*2*_, thus reflecting dishabituation of the motor neuron. This effect is more pronounced for lower values of *g*_*L*_, where the shift in *C*_*2*_ is greater, and the slopes of the BHDH curves are consistently shallower (Fig 10).

As the strength of *C*_*4*_ further increases, the BHDH curves continue to shift to the left with steeper slopes, until the value of *C*_*4*_ is approximately 0.225, where the BHDH curves become vertical, as shown by dashed lines (Fig 10). Beyond this value, waves in the interneuron are unable to propagate to inhibitory synapse *C*_*3*_, resulting in complete dishabituation.

## Discussion

Utilizing a novel approach, we studied variations in the BSH dependences in response to changes of parameters of the reaction-diffusion model with Hebbian type synaptic junctions between neurons. It was found that longer transmembrane potential waves (lower *g*_*L*_), which propagate in the motor neuron, caused the BSH curves to shift towards sensitization. On the contrary, shorter waves (greater *g*_*K*_) triggered the opposite shift of the BSH curves towards habituation. Also, we observed that synaptic strength *C*_*1*_ is another important parameter which has a significant effect on the positioning of BSH.

The value of *C*_*1*_ directly affects the transmembrane potential flux into the interneuron, thus changing its inhibitory influence on the RDMU. Specifically, it was found that different values of *C*_*1*_ either substantially reduced or increased the effectiveness of the inhibitory synapse *C*_*3*_, resulting in a state of the RDMU that is either significantly harder or easier to habituate. In addition to excitatory synapse *C*_*1*_, inhibitory synapse *C*_*4*_ also influences the RDMU through changing conditions for dishabituation. Indeed, just a small increase in *C*_*4*_ produces a notable shift in the BHDH curves towards lower values of excitatory strength in the synapse *C*_*2*_.

There are two possible approaches for incorporating dishabituation in the RDMU. These two approaches can be derived from the existing concepts of superimposition of sensitization and reversal of habituation described in [28]. The first approach is to increase the responsiveness of the motor neuron through an additional strong stimulus, which can be accomplished by adding an additional sensory neuron. In contrast, the second approach is to inhibit the interneuron that causes habituation. While the first approach results in the intertwining of sensitization and dishabituation, since the two processes share the same mechanism, the second one allows sensitization and dishabituation to be further distinguished.

There has been much debate about whether sensitization and dishabituation can be dissociated [28]-[32]. Once again, based on specific experimental procedures, these two processes could either occur through the same mechanisms [29], [30] or could have differing ones [28], [31], [32]. The classic dishabituation described in [4] could be an example of superimposed sensitization, where dishabituation and sensitization both result from direct stimulation to the habituated neuron. However, a more recent revision of this work emphasizes the reversal of habituation as another form of dishabituation [33], which we chose to model using additional input B (Fig 1).

A possible future enhancement to the RDMU would be the ability to model more complex behaviors than described above. Often described alongside sensitization and habituation is training, the process by which a weak input (trained input) becomes able to excite a target neuron by being repeatedly paired with a strong stimulus (training stimulus) [5]. After sufficient stimulations, the strength of the synapses in the trained input increases enough to excite the target neuron independently of the training stimulus. At present, our RDMU is unable to model training because the strengths of synaptic connections are fixed. Additionally, the current RDMU may need to be modified to accommodate series of periodic stimulations, as excitations due to isolated stimuli may not be capable of producing training.

## Supporting information

S1 Appendix **Fig A1**. Numerical mesh used to approximate the Morris-Lecar equations. Each node is one spatial interval *Δx* apart from adjacent nodes. **Fig A2**. Block diagram for solving the explicit grid Eqs. S1 (A1)–S1 (A24). At each time step these equations are solved in the following order: no-flux boundary conditions at the edges, Morris-Lecar equations at the inner grid points, modified-diffusion-term Morris-Lecar equations at the branching node and Hebbian and uni-directional no-flux boundary conditions at the synapses.
(DOCX)
