# Supplementary material for: Reaction-diffusion memory unit: Modeling of sensitization, habituation and dishabituation in the brain
Source: PLoS One. 2019 Dec 5;14(12):e0225169. doi: 10.1371/journal.pone.0225169 (PMC6894767; doi:10.1371/journal.pone.0225169)
Supplement: S1 Appendix — Fig A1. Numerical mesh used to approximate the Morris-Lecar equations. Each node is one spatial interval Δx apart from adjacent nodes. Fig A2. Block diagram for solving the explicit grid Eqs. S1 (A1)–S1 (A24). At each time step these equations are solved in the following order: no-flux boundary conditions at the edges, Morris-Lecar equations at the inner grid points, modified-diffusion-term Morris-Lecar equations at the branching node and Hebbian and uni-directional no-flux boundary conditions at the synapses. (DOCX) [file pone.0225169.s001.docx]

**S1 Appendix: Finite Difference Method**

Matthew M. Carnaghi^1^, Joseph M. Starobin^1*^

^1^ Department of Nanoscience, University of North Carolina at Greensboro, Greensboro, North Carolina, USA; ^*^Corresponding author; E-mail: [jmstarob@uncg.edu](mailto:jmstarob@uncg.edu)

The sensory neuron, input A and motor neurons denoted as cable *N*, the interneuron designated as cable *M*, and input B denoted as cable *P* were discretized using a numerical mesh shown in Fig A1. The temporal part of Eq. (14) was approximated in an explicit manner with a first order finite difference with respect to time, *Δt*. The spatial term of the same equation was approximated using a second order finite difference with respect to spatial interval, *Δx*. Grid Eqs. (14)-(18) were as follows:

$$\begin{aligned} \frac{v_{i}^{n+1}-v_{i}^{n}}{\Delta t}=F_{i}\left( t \right)-g_{L}\left( v_{i}^{n}-v_{L} \right)-M_{i \infty}^{n}g_{Ca}\left( v_{i}^{n}-v_{Ca} \right) \\ -g_{K}w_{i}^{n}\left( v_{i}^{n}-v_{K} \right)+\frac{v_{i+1}^{n}+v_{i-1}^{n}-2v_{i}^{n}}{\Delta x^{2}}\#(A1) \end{aligned}$$

$$\begin{aligned} \frac{w_{i}^{n+1}-w_{i}^{n}}{\Delta t}=\frac{W_{i \infty}^{n}-w_{i}^{n}}{\tau_{i}^{n}}\#\left( A2 \right) \end{aligned}$$

$$\begin{aligned} M_{i \infty}^{n}=\frac{1}{2}\left( 1+\tanh\left( \frac{v_{i}^{n}-v_{1}}{v_{2}} \right) \right)\#\left( A3 \right) \end{aligned}$$

$$\begin{aligned} W_{i \infty}^{n}=\frac{1}{2}\left( 1+\tanh\left( \frac{v_{i}^{n}-v_{3}}{v_{4}} \right) \right)\#\left( A4 \right) \end{aligned}$$

$$\begin{aligned} \tau_{i}^{n}=\frac{1}{\phi}\mathrm{sech} \left( \frac{v_{i}^{n}-v_{3}}{{2v}_{4}} \right)\#\left( A5 \right) \end{aligned}$$

**
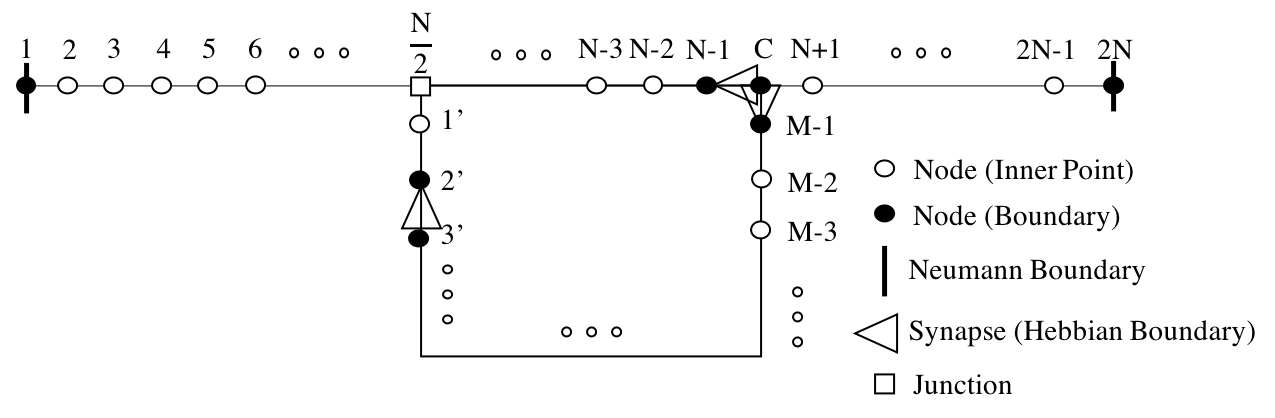
**

**Fig A1**

**Fig A1.** Numerical mesh used to approximate the Morris-Lecar equations. Each node is one spatial interval *Δx* apart from adjacent nodes.

Here *i* is the number of the current spatial node and *n* is the number of the current time step. Eqs. (A1) and (A2) can be re-written to derive the explicit expression for the *n+1* time step using transmembrane potential values from the previous time level:

$$\begin{aligned} v_{i}^{n+1}=v_{i}^{n}+\Delta t\left( \begin{aligned} F_{i}\left( t \right)-g_{L}\left( v_{i}^{n}-v_{L} \right)-M_{i \infty}^{n}g_{Ca}\left( v_{i}^{n}-v_{Ca} \right) \\ -g_{K}w_{i}^{n}\left( v_{i}^{n}-v_{K} \right)+\frac{v_{i+1}^{n}+v_{i-1}^{n}-2v_{i}^{n}}{\Delta x^{2}} \end{aligned} \right)\#\left( A6 \right) \end{aligned}$$

$$\begin{aligned} w_{i}^{n+1}=w_{i}^{n}+\Delta t\left( \frac{W_{i \infty}^{n}-w_{i}^{n}}{\tau_{i}^{n}} \right)\#\left( A7 \right) \end{aligned}$$

These equations can be applied at every inner node except the boundaries, synapses, and branching node *N/2* (Fig A1). The numerical no-flux conditions (Eq. 19) at the boundaries of input A, input B and motor neurons are represented as follows:

$$\begin{aligned} \frac{v_{i+1}^{n}-v_{i-1}^{n}}{2\Delta x}=0\#\left( A8 \right) \end{aligned}$$

Here, *i* is equal to either 1, 1’’ or*2N*. Eq. (A8) provides values of *v* for the extension nodes 0, 0’’ and *2N+1*, which can be added to Eq. (A6) to find the new values of *v* at nodes 1, 1’’ and *2N*:

$$\begin{aligned} v_{0}^{n}=v_{2}^{n}\#\left( A9 \right) \end{aligned}$$

$$\begin{aligned} v_{2N+1}^{n}=v_{2N-1}^{n}\#\left( A10 \right) \end{aligned}$$

$$\begin{aligned} v_{0^{''}}^{n}=v_{2^{''}}^{n}\#\left( A11 \right) \end{aligned}$$

$$\begin{aligned} v_{1}^{n+1}=v_{1}^{n}+\Delta t\left( \begin{aligned} F_{1}\left( t \right)-g_{L}\left( v_{1}^{n}-v_{L} \right)-M_{1 \infty}^{n}g_{Ca}\left( v_{1}^{n}-v_{Ca} \right) \\ -g_{K}w_{1}^{n}\left( v_{1}^{n}-v_{K} \right)+\frac{{2v}_{2}^{n}-2v_{1}^{n}}{\Delta x^{2}} \end{aligned} \right)\#\left( A12 \right) \end{aligned}$$

$$\begin{aligned} v_{2N}^{n+1}=v_{2N}^{n}+\Delta t\left( \begin{aligned} F_{2N}\left( t \right)-g_{L}\left( v_{2N}^{n}-v_{L} \right)-M_{2N \infty}^{n}g_{Ca}\left( v_{2N}^{n}-v_{Ca} \right) \\ -g_{K}w_{2N}^{n}\left( v_{2N}^{n}-v_{K} \right)+\frac{2v_{2N-1}^{n}-2v_{2N}^{n}}{\Delta x^{2}} \end{aligned} \right)\#\left( A13 \right) \end{aligned}$$

$\begin{aligned} v_{1^{''}}^{n+1}=v_{1^{''}}^{n}+\Delta t\left( \begin{aligned} F_{1^{''}}\left( t \right)-g_{L}\left( v_{1^{''}}^{n}-v_{L} \right)-M_{1^{''} \infty}^{n}g_{Ca}\left( v_{1^{''}}^{n}-v_{Ca} \right) \\ -g_{K}w_{1^{''}}^{n}\left( v_{1^{''}}^{n}-v_{K} \right)+\frac{{2v}_{2^{''}}^{n}-2v_{1^{''}}^{n}}{\Delta x^{2}} \end{aligned} \right)\#\left( A14 \right) \end{aligned}$

Using nomenclature from Fig A1, conditions at the synapses given by the Hebbian rule Eq. (20) and a unidirectional no-flux Eq. (21) can be approximated as follows:

$$\begin{aligned} v_{i+1}^{n}=C_{1}\left( v_{N,i}^{n}-v_{o} \right)+C_{4}\left( v_{P,i}^{n}-v_{o} \right)+v_{o}\#\left( A15a \right) \end{aligned}$$

$$\begin{aligned} v_{i+1}^{n}=C_{2}\left( v_{N,i}^{n}-v_{o} \right)+C_{3}\left( v_{M,i}^{n}-v_{o} \right)+v_{o}\#\left( A15b \right) \end{aligned}$$

$$\begin{aligned} v_{i+1}^{n}=C_{2}\left( v_{N,i}^{n}-v_{o} \right)+C_{3}\left( v_{M,i}^{n}-v_{o} \right)+v_{o}\#\left( A15c \right) \end{aligned}$$

$$\begin{aligned} 3v_{i}^{n}-4v_{i-1}^{n}+v_{i-2}^{n}=0\#\left( A16 \right) \end{aligned}$$

where *i* and *i+1* are the pre-synaptic and post-synaptic nodes of each synapse, respectively. Eq. (A16) has a second order approximation with respect to the spatial interval *Δx*. Eqs. (A15), (A16) provide the unknown values of synaptic and post-synaptic potentials as follows:

$$\begin{aligned} v_{2^{'}}^{n}=\frac{4v_{1^{'}}^{n}-v_{\frac{N}{2}}^{n}}{3}\#\left( A17 \right) \end{aligned}$$

$$\begin{aligned} v_{N-1}^{n}=\frac{4v_{N-2}^{n}-v_{N-3}^{n}}{3}\#\left( A18 \right) \end{aligned}$$

$$\begin{aligned} v_{M-1}^{n}=\frac{4v_{M-2}^{n}-v_{M-3}^{n}}{3}\#\left( A19 \right) \end{aligned}$$

$$\begin{aligned} v_{P}^{n}=\frac{4v_{P-1}^{n}-v_{P-2}^{n}}{3}\#\left( A20 \right) \end{aligned}$$

$$\begin{aligned} v_{3^{'}}^{n}=C_{1}\left( \frac{4v_{1^{'}}^{n}-v_{\frac{N}{2}}^{n}}{3}-v_{o} \right)+C_{4}\left( \frac{4v_{P-1}^{n}-v_{P-2}^{n}}{3}-v_{o} \right)+v_{o}\#\left( A21 \right) \end{aligned}$$

$$\begin{aligned} v_{C}^{n}=C_{2}\left( \frac{4v_{N-2}^{n}-v_{N-3}^{n}}{3}-v_{o} \right)+C_{3}\left( \frac{4v_{N-2}^{n}-v_{M-3}^{n}}{3}-v_{o} \right)+v_{o}\#\left( A22 \right) \end{aligned}$$

At the branching node *N/2* (Eq. (22)) we add two one-dimensional diffusion terms from

Eq. (A6) to account for diffusion in both branches. According to Eq. (22), the summed diffusion

terms as well as a modified Eq. A6 at the node *N/2* are given as follows:

$$\begin{aligned} \frac{\partial^{2}v}{\partial x^{2}}+\frac{\partial^{2}v}{\partial y^{2}}\to\frac{v_{\frac{N}{2}-1}^{n}+v_{\frac{N}{2}+1}^{n}-2v_{\frac{N}{2}}^{n}}{\Delta x^{2}}+\frac{v_{1^{'}}^{n}+v_{\frac{N}{2}-1}^{n}-2v_{\frac{N}{2}}^{n}}{\Delta y^{2}}, \Delta y=\Delta x\#\left( A23 \right) \end{aligned}$$

$$\begin{aligned} v_{\frac{N}{2}}^{n+1}=v_{\frac{N}{2}}^{n}+\Delta t\left( \begin{aligned} F_{\frac{N}{2}}\left( t \right)-g_{L}\left( v_{\frac{N}{2}}^{n}-v_{L} \right)-M_{\frac{N}{2} \infty}^{n}g_{Ca}\left( v_{\frac{N}{2}}^{n}-v_{Ca} \right) \\ -g_{K}w_{\frac{N}{2}}^{n}\left( v_{\frac{N}{2}}^{n}-v_{K} \right)+\frac{{2v}_{\frac{N}{2}-1}^{n}+v_{1^{'}}^{n}+v_{\frac{N}{2}+1}^{n}-{4v}_{\frac{N}{2}}^{n}}{\Delta x^{2}} \end{aligned} \right)\#\left( A24 \right) \end{aligned}$$

The algorithm for solving the mesh Eqs. (A1) – (A24) is demonstrated in Fig A2. At every *n^th^* time level the algorithm starts with application of the no-flux boundary conditions to determine values of $v$ at the extended nodes of the sensory and motor neurons. After that Eq. (A6) is solved to determine values of $v$ for all inner points at the *(n+1)^th^* time level. Then, Eq. (A24) is used to compute $v$ at the *(n+1)^th^* time level at the branching node. Finally, the *(n+1)^th^* values of $v$ can be determined at synaptic and post-synaptic nodes using Eqs. (A17)-(A22). Once Eqs. (A1) – (A24) have been solved at the *(n+1)^th^* time level for all nodes, values of *v^n+1^* are redefined as ones at the previous *n^th^* level, the process is repeated.

**
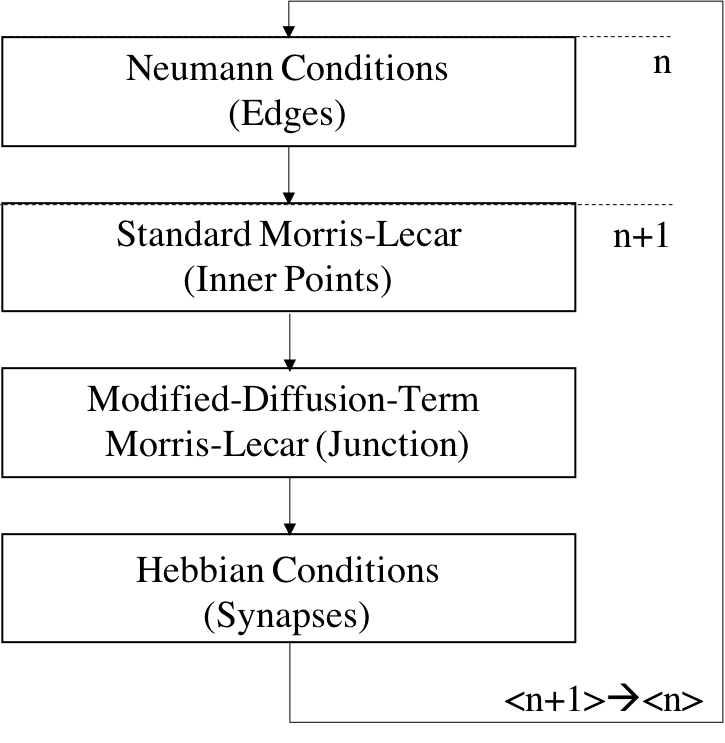
**

**Fig A2**

**Fig A2.** Block diagram for solving the explicit grid Eqs. (A1) – (A24). At each time step these equations are solved in the following order: no-flux boundary conditions at the edges, Morris-Lecar equations at the inner grid points, modified-diffusion-term Morris-Lecar equations at the branching node and Hebbian and uni-directional no-flux boundary conditions at the synapses.
